# Consumption of fructose- but not glucose-sweetened beverages for 10 weeks increases circulating concentrations of uric acid, retinol binding protein-4, and gamma-glutamyl transferase activity in overweight/obese humans

**DOI:** 10.1186/1743-7075-9-68

**Published:** 2012-07-24

**Authors:** Chad L Cox, Kimber L Stanhope, Jean Marc Schwarz, James L Graham, Bonnie Hatcher, Steven C Griffen, Andrew A Bremer, Lars Berglund, John P McGahan, Nancy L Keim, Peter J Havel

**Affiliations:** 1 Department of Molecular Biosciences, School of Veterinary Medicine, University of California, Davis, One Shields Avenue, Davis, CA, 95616, USA; 2 Department of Nutrition, University of California, Davis, One Shields Ave, Davis, CA, 95616, USA; 3 Department of Basic Sciences, College of Osteopathic Medicine, Touro University, 1310 Johnson Lane, Mare Island, Vallejo, CA, 94592, USA; 4 Department of Internal Medicine, UCD School of Medicine, 4610 X St., Sacramento, CA, 95817, USA; 5 Department of Pediatrics, School of Medicine, Vanderbilt University, Nashville, TN, 37232, USA; 6 United States Department of Agriculture, Western Human Nutrition Research Center, 430 W. Health Sciences Dr., Davis, CA, 95616, USA; 7 Department of Radiology, UCD Medical Center, 2315 Stockton Blvd., Sacramento, CA, 95817, USA

## Abstract

**Background:**

Prospective studies in humans examining the effects of fructose consumption on biological markers associated with the development of metabolic syndrome are lacking. Therefore we investigated the relative effects of 10 wks of fructose or glucose consumption on plasma uric acid and RBP-4 concentrations, as well as liver enzyme (AST, ALT, and GGT) activities in men and women.

**Methods:**

As part of a parallel arm study, older (age 40–72), overweight and obese male and female subjects (BMI 25–35 kg/m^2^) consumed glucose- or fructose-sweetened beverages providing 25% of energy requirements for 10 wks. Fasting and 24-h blood collections were performed at baseline and following 10 wks of intervention and plasma concentrations of uric acid, RBP-4 and liver enzyme activities were measured.

**Results:**

Consumption of fructose, but not glucose, led to significant increases of 24-h uric acid profiles (*P* < 0.0001) and RBP-4 concentrations (*P* = 0.012), as well as plasma GGT activity (*P* = 0.04). Fasting plasma uric acid concentrations increased in both groups; however, the response was significantly greater in subjects consuming fructose (*P* = 0.002 for effect of sugar). Within the fructose group male subjects exhibited larger increases of RBP-4 levels than women (*P* = 0.024).

**Conclusions:**

These findings suggest that consumption of fructose at 25% of energy requirements for 10 wks, compared with isocaloric consumption of glucose, may contribute to the development of components of the metabolic syndrome by increasing circulating uric acid, GGT activity, suggesting alteration of hepatic function, and the production of RBP-4.

## Background

Between 1970 and 2005 there has been a 19% increase in the consumption of added sugars and sweeteners in the U.S. [1]. Current estimations suggest that sweetener consumption in the U.S. has increased to an average of 477 kcal/person, or approximately 24% of a typical 2000 kcal/day diet [1]. The monosaccharide fructose is a primary component of both of the two most common sweeteners in the U.S., sucrose and high fructose corn syrup. An increase in the consumption of sweeteners containing fructose has occurred in parallel with the increasing prevalence of overweight and obesity over the past three decades [2], suggesting that increased consumption of fructose may contribute to the current epidemic of obesity-related metabolic disorders [2,3] including increased incidence of the metabolic syndrome [4,5].

Recently we reported that consumption of fructose-sweetened beverages for 10 wks, at 25% of energy requirements, increases several risk factors for the metabolic syndrome in older (age 40–72), overweight/obese humans (BMI 25–35 kg/m^2^) as compared to isocaloric glucose consumption [6]. Consumption of fructose, but not glucose led to increased hepatic *de novo* lipogenesis (DNL), and promoted accumulation of intra-abdominal fat, the development of a more atherogenic lipid profile, and reduced insulin sensitivity in overweight/obese adults [6]. We have hypothesized that the increased rate of DNL following consumption of fructose leads to an accumulation of hepatic lipid, which promotes dyslipidemia and decreased insulin sensitivity [7].

Evidence suggests that elevated levels of uric acid and increased activity of the liver enzymes gamma-glutamyl transferase (GGT) and alanine aminotransferase (ALT) are also associated with development of the metabolic syndrome [8,9]. Fructose has been demonstrated to increase circulating uric acid concentrations in both animals and humans, and elevated concentrations of uric acid are correlated with increased fructose intake [10,11]. It has been suggested that fructose-induced hyperuricemia may mediate some of the abnormalities associated with metabolic syndrome including hypertriglyceridemia, insulin resistance and hypertension [10]. Elevated serum uric acid levels have also been found to be associated with the development of cirrhosis and increased plasma activity of GGT and ALT [12]. GGT and ALT activities (and to a lesser extent aspartate aminotransferase (AST)) have been shown to be strong predictors of metabolic syndrome and are considered to be markers of non-alcoholic fatty liver disease (NAFLD) [13,14]. Uric acid levels have also been found to be strongly associated with concentrations of the recently described adipokine retinol binding protein-4 (RBP-4) [15]. Increased circulating concentrations of RBP-4 have been linked with increased visceral adiposity [16] and have been shown to directly contribute to hepatic insulin resistance via induction of hepatic glucose production [17] and impairment of insulin signaling in muscle [15,18]. To determine if fructose-induced changes of DNL, intra-abdominal fat deposition, circulating lipids and insulin sensitivity [6] were accompanied by increases of these biological markers associated with the development of metabolic syndrome, 24-h plasma uric acid levels as well as fasting concentrations of RBP-4 and activities of the liver enzymes GGT, AST and ALT were measured in overweight/obese men and women consuming fructose- or glucose-sweetened beverages for 10 wks.

## Methods

### Study design

This was a parallel arm study with 3 phases: 1) a 2-wk inpatient baseline period; 2) an 8-wk outpatient intervention period; and 3) a 2-wk inpatient intervention period. During baseline, subjects resided in the University of California-Davis Clinical and Translational Science Center’s Clinical Research Center (CCRC) for 2 wks. Subjects then began the 8-wk outpatient intervention and consumed either fructose- (n = 17) or glucose-sweetened (n = 15) beverages at 25% of energy requirements with self-selected *ad libitum* diets. Subjects returned to the CCRC for the final 2 wks of intervention during which the glucose- or fructose-sweetened beverages were consumed as part of an energy-balanced diet.

### Subjects

Participants were recruited through newspaper advertisements and underwent a telephone and an in-person interview with medical history, a complete blood count, and a serum biochemistry panel to assess eligibility. Inclusion criteria included age 40–72 years and BMI 25–35 kg/m^2^ with a self-report of stable body weight during the prior six months. Women were post-menopausal based on a self-report of no menstruation for at least one year. Exclusion criteria included: evidence of diabetes, renal or hepatic disease, fasting serum triglyceride (TG) concentrations >400 mg/dl, hypertension (>140/90 mm Hg), and surgery for weight loss. Individuals who smoked, reported exercise of more than 3.5 hours/wk at a level more vigorous than walking, or having used thyroid, lipid-lowering, glucose-lowering, anti-hypertensive, anti-depressant, or weight loss medications were also excluded. Diet-related exclusion criteria included habitual ingestion of more than one sugar-sweetened beverage/day (12 ounces or ~350 mL) or more than two alcoholic beverages/day (one alcoholic beverage is defined as 5 ounces of wine, 12 ounces of beer, 8 ounces of malt liquor, or 1.5 ounces of spirits (80 proof) which is equivalent to 14 grams or 0.6 ounces of ethanol). The UCD Institutional Review Board approved the experimental protocol, and subjects provided informed consent to participate in the study. Thirty-nine subjects enrolled in the study and experimental groups were matched for gender, BMI, and fasting TG and insulin concentrations. As reported by Stanhope et al., baseline anthropometric and metabolic characteristics did not differ between the two experimental groups (Table 1) [6]. Seven subjects (3 in the glucose group, 4 in the fructose group) did not complete the study because of inability/unwillingness to comply with the protocol or due to personal or work-related conflicts. We were unable to measure uric acid, liver enzymes or RBP-4 in one female subject in the fructose group due to a lack of plasma samples. We were also unable to collect a complete set of 24-h blood samples from one subject in the glucose group. Therefore, 24-h uric acid exposure was calculated with n = 30 (fructose: n = 16, glucose: n = 14), while all other measurements were calculated with n = 31 (fructose: n = 16, glucose: n = 15).

**Table 1:** Baseline anthropometric & metabolic parameters

**Parameter**	**Glucose**		**Fructose**	
	**Male (n = 7)**	**Female (n = 8)**	**Male (n = 9)**	**Female (n = 8)**
Age (yr)	54 ± 3	56 ± 2	52 ± 4	53 ± 2
Weight (kg)	88.4 ± 2.9	84.0 ± 4.5	89.3 ± 2.9	81.9 ± 4.2
BMI (kg/m^2^)	29.3 ± 1.1	29.4 ± 1.3	28.4 ± 0.7	30.3 ± 1.0
Waist circumference (cm)	98.9 ± 2.6	91.0 ± 4.0	97.3 ± 3.3	91.8 ± 4.4
Body Fat (%)	29.4 ± 1.1	43.2 ± 1.5	28.5 ± 1.3	39.6 ± 2.2
TG (mg/dl)	148 ± 31	145 ± 23	131 ± 21	159 ± 30
Total Cholesterol (mg/dl)	179 ± 14	193 ± 10	176 ± 6	198 ± 15
HDL (mg/dl)	36 ± 3	41 ± 3	39 ± 4	41 ± 3
LDL (mg/dl)	124 ± 5	123 ± 11	107 ± 7	124 ± 15
Glucose (mg/dl)	89 ± 2	89 ± 3	88 ± 1	90 ± 1
Insulin (μU/ml)	14.3 ± 3.2	15.6 ± 2.9	12.0 ± 1.6	16.3 ± 2.5

GLM 2-factor ANOVA (type of sugar & gender). There were no significant differences among groups. Data represent mean ± SEM.

Data previously published: Stanhope KL, *et al.* (2009). *J Clin Invest* 119: 1322–1334.

### Diets

During the inpatient metabolic phases, subjects consumed diets designed to maintain energy balance providing 15% of energy as protein, 30% as fat, and 55% as carbohydrate. Subjects were required to consume all of the food and were limited to only the food provided. Daily energy intake was based on an estimate of energy needed to maintain body weight, calculated at baseline using the Mifflin equation to estimate resting energy expenditure [19] and adjusted for activity using a multiplication factor of 1.3 on the day of 26-h stable isotope infusions and 24-h serial blood collections, and a factor of 1.5 for other inpatient days. Energy intake was distributed across the day: 25% at breakfast (09:00 h), 35% at lunch (13:00 h), and 40% at dinner (18:00 h). During baseline, the carbohydrate content consisted primarily of complex carbohydrates. For the final 2-wk inpatient intervention period, subjects consumed diets at the baseline energy level and macronutrient composition except that 30% of energy was from complex carbohydrates and 25% was provided by fructose- or glucose-sweetened beverages. The macronutrient and micronutrient content of the diets consumed by the two experimental groups during the inpatient baseline and inpatient intervention phases of the study was identical, with the exception of the sugars provided in the beverages. Additional details about the dietary intake for inpatient and outpatient phases have been described previously [6].

### Meals consumed during and prior to 24-h blood collections

Intervention meals (10 wk) were matched as closely as possible to the baseline meals (0 wk), except for the substitution of 25% of energy from sugars for the complex carbohydrate. The baseline (0 wk) and final (10 wk) intervention 24-h blood collections were performed after subjects had consumed energy-balanced, weight-maintaining diets in the CCRC for 10 days.

### 24-hour fasting and postprandial blood profiles

24-h blood collections were conducted during baseline (0 wk) and after 10 wks of intervention (10 wk). At 07:30 h, an i.v. catheter was inserted into an arm vein by a Registered Nurse and kept patent with slow saline infusion. Three fasting blood samples were collected in EDTA at 08:00, 08:30, and 09:00 h. Thirty-three postprandial blood samples were collected at 30–60 minute intervals from 09:30 until 08:00 h the next morning [20,21]. Meals were served at 09:00, 13:00 and 18:00 h. An additional 3–6 ml of blood was collected at each of the following time-points: 08:00, 08:30, 09:00 and 22:00, 23:00, 23:30 h. The plasma from the 3 fasting samples (08:00, 08:30, 09:00 h) was pooled, as was the plasma from the 3 postprandial blood samples (22:00, 23:00, 23:30 h); multiple aliquots of each pooled sample were stored at −80 °C.

### Uric Acid, RBP-4, GGT, AST and ALT

Uric Acid was measured using a colorimetric assay from Wako (Richmond, VA). RBP-4 was quantified using an ELISA from R&D Systems (Minneapolis, MN) and GGT, AST and ALT activities were determined using a Polychem Chemistry Analyzer (PolyMedCo, Inc., Cortlandt Manor, NY).

### Data analysis

Average 24-h uric acid and TG exposure were determined by averaging values for the 33 postprandial time periods. Peak TG exposure was defined as the highest TG measurement over the 33 postprandial time periods, and postprandial TG peak was calculated by averaging TG measurements taken at 22:00, 23:00, and 23:30 h. Statistical tests were performed with SAS 9.2. The percent change for each outcome was calculated and analyzed in a 3-factor (type of sugar, gender, and presence of metabolic syndrome) mixed procedures analysis using PROC MIXED. Outcomes with least squares means (LS means) of the change (10wk versus 0wk) significantly different than zero were identified. Risk factors for metabolic syndrome (MSRFs) were identified in all subjects and the presence of metabolic syndrome was defined as having at least 3 MSRFs (subjects with metabolic syndrome: Fructose n = 5, Glucose n = 4; subjects without metabolic syndrome: Fructose n = 11, Glucose n = 11). MSRFs were those defined by the American Heart Association/National Heart Lung and Blood Institute [22,23]. The percent change in response variables between 0 and 10 wks was determined, and Pearson’s correlation coefficients describing the relationship between response variables were calculated using PROC CORR. Statistical tests with *P* values <0.05 were considered significant. Data are presented as mean ± SEM.

## Results

### Fasting uric acid and 24-h circulating uric acid profiles

**Figure 1:**
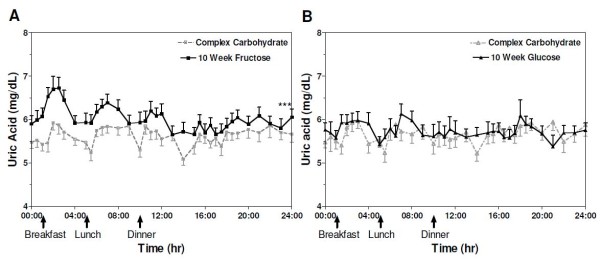
**Circulating uric acid concentrations over 24-h in subjects before and after 10 wks of consuming (A) fructose- or (B) glucose- sweetened beverages.** ***P < 0.0001 compared with baseline and *P* < 0.0001 for effect of sugar using PROC mixed 3-factor (sugar, gender, and presence of metabolic syndrome) ANOVA; glucose, n = 14; fructose, n = 16. Data represent mean ± SEM.

At 10 wks of intervention fasting plasma uric acid concentrations were significantly increased from baseline in subjects consuming both fructose- (absolute Δ = +0.82 ± 0.08 mg/dL; *P* < 0.0001) and glucose-sweetened (absolute Δ = +0.23 ± 0.09 mg/dL; *P* = 0.02) beverages, but the effect was significantly greater in those consuming fructose-sweetened beverages (*P* = 0.002 for effect of sugar) (Table 2). 24-h serum uric acid profiles were increased significantly from baseline in subjects consuming fructose (absolute Δ = +0.42 ± 0.05 mg/dL; *P* < 0.0001) but not in those consuming glucose (absolute Δ = +0.07 ± 0.03 mg/dL; *P* = 0.26) (*P* < 0.0001 for effect of sugar) (Table 2) (Figure 1).

**Table 2:** **Baseline values and % change at 10 wks of intervention for fasting uric acid levels, mean 24-h uric acid exposure, fasting plasma RBP-4 concentrations, and fasting GGT, ALT and AST activities**^**1**^

	**Fructose Baseline**	**Fructose 10 wks**	**Fructose % change**	**Glucose Baseline**	**Gluctose 10 wks**	**Glucose % change**	***P*-value for effect of sugar**
**24-h Uric Acid Exposure (mg/dL)**	5.64 ± 0.14	6.05 ± 0.16	7.2 ± 1.1***	5.67 ± 0.18	5.74 ± 0.18	1.3 ± 1.1	< 0.0001
**Fasting Uric Acid (mg/dL)**	5.70 ± 0.11	6.40 ± 0.13	11.9 ± 0.6***	5.42 ± 0.16	5.65 ± 0.18	4.3 ± 1.5*	0.002
^**2**^**RBP-4 (ug/mL)**	23.0 ± 1.1	25.2 ± 1.5	9.9 ± 4.1*	28.2 ± 1.4	24.5 ± 1.3	−12.5 ± 3.0**	< 0.0001
^**3**^**GGT (U/L)**	23.0 ± 4.1	26.7 ± 4.4	17.7 ± 5.2*	40.2 ± 9.6	33.8 ± 7.6	−10.7 ± 4.0***	< 0.0001
**AST (U/L)**	17.3 ± 1.6	16.1 ± 1.2	−3.7 ± 4.9	22.3 ± 2.3	17.8 ± 1.0	−13.1 ± 6.2**	0.08
**ALT (U/L)**	14.5 ± 1.6	13.7 ± 1.8	−5.2 ± 4.4	20.6 ± 3.2	13.7 ± 1.2	−22.3 ± 6.7 **	0.04

^1^Values are means ± SEM, n = 31 (fructose n = 16; glucose n = 15) for all measurements except 24-h uric acid exposure (n = 30, fructose n = 16, glucose n = 14). PROC MIXED 3-factor (sugar, gender, and presence of metabolic syndrome) ANOVA; ^2^sugar × gender: *P* =0.037; ^3^effect of metabolic syndrome: *P* = 0.002, sugar × metabolic syndrome: *P* = 0.002.

*P < 0.05, **P < 0.01, ***P < 0.001 for changes significantly different from zero.

### Fasting RBP-4

**Figure 2:**
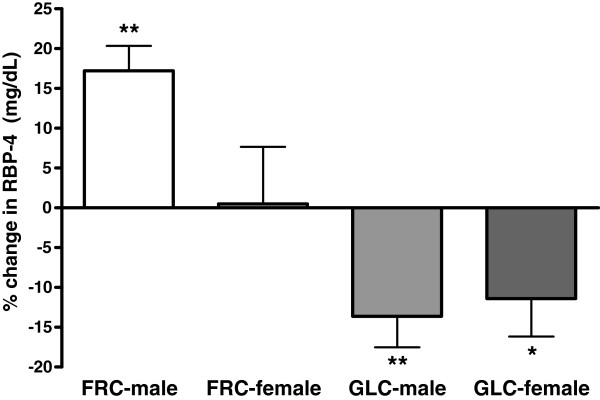
**Percent changes of fasting RBP-4 concentrations after 10 wks of consuming fructose- or glucose-sweetened beverages in male and female subjects.** Values are means ± SEM, n = 31 (fructose group, n = 16; glucose group, n = 15) PROC MIXED 3-factor (sugar, gender, and presence of metabolic syndrome) ANOVA; *P < 0.05 and **P < 0.01 for changes significantly different from baseline.

After 10 wks fasting plasma RBP-4 concentrations increased significantly from baseline in subjects consuming fructose-sweetened beverages (absolute Δ = +2.2± 1.0 ng/mL; *P* = 0.012) and decreased significantly from baseline in those consuming glucose-sweetened beverages (absolute Δ = −3.7 ± 0.9 ng/mL; *P* = 0.0005) (*P* < 0.0001 for effect of sugar) (Table 2). Although RBP-4 concentrations decreased comparably in both men and women in the glucose group, the increase of RBP-4 from baseline levels in subjects consuming fructose was significantly greater in men (*P* = 0.007) compared to women (*P* = 0.908) (*P* = 0.024 for effect of sugar × gender) (Figure 2).

### Plasma GGT, AST, and ALT Activities

Plasma GGT activity was significantly elevated compared with baseline values following 10 wks of fructose consumption (absolute Δ = +3.7 ± 1.1 U/L; *P* = 0.04) but decreased significantly in subjects consuming glucose (absolute Δ = −6.4 ± 2.6 U/L; *P* < 0.0001)(*P* < 0.0001 for effect of sugar) (Table 2). The increases of GGT at 10 wks in subjects consuming fructose were greater in those with less than 3 MSRFs (Baseline: 23.0 ± 5.1 U/L, absolute Δ = +4.1 ± 1.4 U/L) as compared to those with metabolic syndrome (≥3 MSRFs) (Baseline: 23.1 ± 7.3 U/L, absolute Δ = +2.7 ± 1.9 U/L) (*P* = 0.002 for effect of MSRF and P = 0.002 for effect of sugar × MSRF). Fasting activities of AST and ALT decreased slightly following 10 wks of fructose consumption; however, these changes were not statistically significant (Table 2). In subjects consuming glucose both AST (absolute Δ = −4.5 ± 2.2 U/L, *P* = 0.002; effect of sugar: *P* = 0.08) and ALT (absolute Δ = −6.9 ± 3.0 U/L, *P* = 0.002; effect of sugar: *P* = 0.04) activities were decreased after 10 wks (Table 2).

### Relationships between response variables

**Figure 3:**
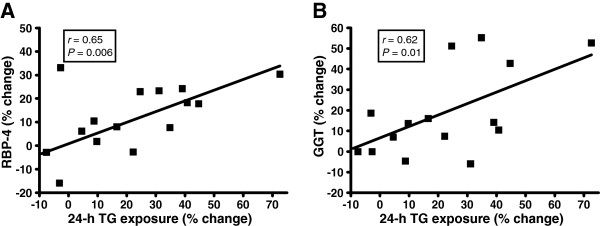
**Correlations between the percent changes of 24-h TG exposure and percent changes of (A) RBP-4 levels and (B) GGT activity in subjects consuming fructose**. Pearson’s correlation coefficients (r) were calculated using PROC CORR. Average 24-h TG exposure was determined as the mean of TG concentrations in the 33 postprandial samples. n = 31 (fructose group n = 16).

In subjects consuming fructose the percent increases of both fasting concentrations of RBP-4 and GGT activity, but not uric acid concentrations, were positively associated with previously reported increases of TG exposure, peak TG exposure, and postprandial TG peak (Table 3) (Figure 3) [6]. These relationships were not observed in subjects consuming glucose (Table 3). There were no significant relationships observed between elevations of uric acid and RBP-4 levels or GGT activity, nor between elevations of RBP-4 levels and GGT activity.

**Table 3:** Correlations between selected response variables (% change)

**Response variables; percent change**	**Fructose (n = 16)**	**Glucose (n = 15)**
RBP-4 vs. peak TG exposure	*r* = 0.719	*r* = −0.011
	*P* = 0.002**	*P* = 0.969
GGT vs. peak TG exposure	*r* = 0.498	*r* = 0.152
	*P* = 0.049*	*P* = 0.603
RBP-4 vs. 24-h TG exposure	*r* = 0.653	*r* = 0.081
	*P* = 0.006**	*P* = 0.783
GGT vs. 24-h TG exposure	*r* = 0 .618	*r* = 0.072
	*P* = 0.011*	*P* = 0.806
RBP-4 vs. postprandial TG peak	*r* = 0.575	*r* = −0.055
	*P* = 0.020*	*P* = 0.851
GGT vs. postprandial TG peak	*r* = 0.601	*r* = 0.178
	*P* = 0.014*	*P* = 0.542

Pearson’s correlation coefficients (*r*) describing the relationship between response variables (% change) were calculated using PROC CORR. Average 24-h TG exposure was determined by averaging values for the 33 postprandial time periods. Peak TG exposure was defined as the highest TG measurement over the 33 postprandial time periods, and postprandial TG peak was calculated by averaging TG measurements taken at 22:00, 23:00, and 23:30 h. *P < 0.05 ** *P* < 0.01.

## Discussion

To our knowledge, this is the first study to report that prolonged fructose consumption increases 24-h circulating uric acid concentrations, GGT activity and RBP-4 levels in humans. Importantly, these findings also complement our previously reported results (in these same subjects) which demonstrated that hepatic fractional *de novo* lipogenesis (DNL) and plasma lipids and lipoprotein concentrations increased in subjects consuming fructose-sweetened beverages, but remained unchanged in subjects consuming glucose-sweetened beverages for 10 wks [6]. Despite comparable weight gain (~1-2% of initial body weight), subjects consuming fructose primarily exhibited increases of visceral adipose tissue (VAT), whereas only subcutaneous adipose tissue (SAT) was increased in subjects consuming glucose [6]. In addition, indices of insulin sensitivity and glucose tolerance were decreased during 10 wks of fructose consumption, but were unaffected by isocaloric consumption of glucose [6].

### Uric acid

Prolonged fructose consumption significantly increased both fasting uric acid concentrations and 24-h uric acid exposure. The increase of fasting uric acid concentrations exhibited after 10 wks of fructose consumption is consistent with previous reports of the effects of up to 5 wks of fructose consumption at 20-30% of total calories in humans [24-26]. Reiser et al. demonstrated that consumption of 20% of energy from fructose for 5 wks as part of a diet designed to maintain body weight increased fasting uric acid levels in men [25] and our results support these findings.

The mechanism by which fructose consumption leads to increased uric acid concentrations is thought to be initiated by the depletion of adenosine triphosphate (ATP) and inorganic phosphate (Pi) resulting from unregulated production of fructose-1-phosphate and glyceraldehyde-3-phosphate from fructose, which bypasses the rate-limiting step of glycolysis, phosphofructokinase (PFK) [3,27]. Fructose-induced depletion of ATP and Pi leads to a concomitant increase of purine nucleotide degradation and, subsequently, uric acid production [27]. It has also been reported that, in addition to increased nucleotide degradation, prolonged fructose consumption promotes increases in the incorporation of glycine into urate suggesting that upregulation of *de novo* purine nucleotide synthesis may also contribute to fructose-induced increases of uric acid [28]. While uric acid is a potent and physiologically relevant antioxidant, recent evidence suggests that elevated levels may be associated with increased oxidative stress, and there is still considerable debate as to which of these roles is more important in the context of metabolic disease [29].

Elevations of fasting uric acid levels have been shown to be associated with hypertriglyceridemia and insulin resistance [30,31]. Our results are not consistent with these findings as we did not detect any relationships between changes in fasting uric acid levels or 24-h uric acid exposure and the previously reported marked increases of TG exposure or reductions of insulin sensitivity in subjects consuming fructose [6]. Also in contrast to the findings of previous investigations [12,15], increases of fasting uric acid concentrations and 24-h uric acid exposure were not associated with elevations of RBP-4 levels or GGT activity. Although we did not detect a correlation between elevations of uric acid and changes of TG exposure, insulin sensitivity, RBP-4 levels or GGT activity, it is important to point out that our sample size was fairly modest and thus these findings do not rule a contribution of uric acid to the reported changes in these metabolic parameters. Additional intervention studies are needed to investigate the possibility that these changes are related. It is interesting that fasting uric acid concentrations (but not 24-h uric acid exposure) also increased significantly in subjects consuming glucose-sweetened beverages for 10 wks despite the fact that these subjects did not exhibit any of the adverse changes measured in subjects consuming fructose [6]. This finding suggests that changes of 24-h uric acid exposure may be a more sensitive indicator of increased metabolic dysfunction than changes of fasting levels.

### RBP-4

No previous studies have investigated the effects of glucose or fructose consumption on circulating levels of RBP-4. There is increasing evidence suggesting that RBP-4 may be an important link between increases of visceral adiposity and insulin resistance [32]. In animal studies RBP-4 has been clearly shown to reduce glucose uptake and impair insulin signaling in muscle, as well as to increase hepatic glucose production via induction of the gluconeogenic enzyme phosphoenolpyruvate carboxykinase (PEPCK) [17]. In humans, increases of circulating RBP-4 are strongly associated with insulin resistance in adipose tissue [33], and elevations of circulating RBP-4 are predictive of a diagnosis of metabolic syndrome [34]. While hepatocytes are the primary source of RBP-4, it has been demonstrated that adipocytes can also contribute significantly to circulating concentrations of RBP-4 [35]. Reduced expression of the insulin-stimulated glucose transporter, GLUT4, in adipocytes has been shown to lead directly to increased adipocyte secretion of RBP-4 [33]. Moreover, expression of RBP-4 in humans is significantly greater in visceral adipose tissues (VAT) compared with subcutaneous adipose tissue (SAT) and is associated with an increase of adipocyte size [16].

Circulating RBP-4 concentrations are significantly elevated following 10 wks of fructose consumption (Table 2) and these changes are correlated with increases of postprandial TG (Table 3, Figure 3). We have hypothesized that fructose consumption can promote reductions in insulin sensitivity by providing substrate for hepatic DNL leading to hepatic triglyceride accumulation, PKC activation, and increased hepatic insulin resistance [7] and have suggested that this mechanism is responsible for the reductions in insulin sensitivity previously reported in these same subjects [6]. Although the reported increases of RBP-4 were relatively modest, our results suggest the possibility that fructose-induced increases of RBP-4 levels may also have contributed to the previously reported reductions of insulin sensitivity in these subjects [6].

We have suggested that the differential effects of fructose and glucose on regional adipose deposition may be explained in part by the increased sensitivity of SAT relative to VAT to insulin-stimulated lipoprotein lipase (LPL) activation [6], and by our reported observations that insulin responses were decreased in subjects consuming fructose and increased in subjects consuming glucose [36]. The differential changes in RBP-4 levels in subjects consuming fructose or glucose are consistent with this mechanism considering that reductions of post-meal insulin exposure in subjects consuming fructose would lead to decreased expression of GLUT4 in adipocytes, which would be expected to be associated with an increase in the production and secretion of RBP-4 from adipose tissue (VAT in particular). Thus, it is possible that reduced insulin exposure in subjects consuming fructose led to increased plasma RBP-4 levels directly by decreasing expression of GLUT4 in adipose tissue, and indirectly by increasing deposition of TG into VAT, leading to increased visceral adipocyte size, and increased secretion of RBP-4. The significant reduction of circulating RBP-4 concentrations observed in subjects consuming glucose-sweetened beverages is consistent with the observations that glucose consumption did not result in increased DNL, postprandial hypertriglyceridemia, accumulation of VAT or reduced postprandial insulin exposure [6].

The finding that increases of RBP-4 during fructose consumption were larger in men than in women was not unexpected considering that TG responses and increases of VAT deposition were also considerably greater in men [6] (Figure 2). We have also reported that women exhibited greater decreases in insulin sensitivity than men in response to fructose consumption, and have hypothesized that this may be due to decreased rates of VLDL production and secretion, leading to larger increases of hepatic lipid [6]. Our findings suggest that the increased rate of VLDL production/secretion following fructose consumption in men is accompanied by an increase of RBP-4 levels. These elevations of RBP-4 levels are likely the result of increased accumulation of VAT and decreased insulin-stimulated GLUT4 expression in adipocytes. These data suggest that in men fructose consumption likely contributes to increases of hepatic insulin resistance both directly by providing substrate for hepatic DNL leading to increased hepatic TG accumulation and decreased hepatic insulin sensitivity [7], and indirectly by increasing visceral adiposity [37], while in women it is primarily a direct effect mediated by increased hepatic DNL and lipid content.

### Liver enzymes

There have been very few prospective investigations of the effects of fructose consumption on the activity of the liver enzymes GGT, AST and ALT in humans. However, the results from one recent study suggest that short-term fructose overfeeding does not alter AST or ALT activities [38], and our results following 10 wks of fructose consumption are in agreement with these findings. Reports on the effects of fructose consumption on GGT activity in humans are not available, and the results of animal studies are not likely to be physiologically relevant since these investigations have primarily been conducted rats, which have been reported to have hepatic and plasma GGT activities over 20-fold lower than humans [39]. The results of the present study demonstrate that prolonged fructose consumption leads to marked increases of GGT activity in older, overweight/obese adults (Table 2).

We also report that fructose-induced increases of plasma GGT activity are positively associated with increases of 24-h TG exposure, peak TG exposure and the postprandial TG peak reported previously in these same subjects [6] (Table 3, Figure 3). Martin *et al.* first demonstrated that GGT activity is strongly associated with postprandial plasma TG levels [40], which has since been confirmed by other investigators [41]. The authors hypothesized that the upregulation of hepatic microsomal enzymes that results in increased TG synthesis and DNL is accompanied by a concomitant increase in GGT activity and speculated that this process could be initiated by excessive carbohydrate intake [40]. The increases of GGT activity in subjects consuming fructose support the mechanism proposed by Martin *et al*. However, since GGT activity significantly decreased during 10 wks of glucose consumption, and these changes were not related to measures of TG exposure, we suggest that, under energy balanced conditions, it is not carbohydrate in general (as speculated by Martin *et al.*) but rather intake of fructose specifically that mediates increases of hepatic DNL, TG synthesis, and GGT activity.

GGT has been well established as a reliable marker of increased hepatic lipid content and hepatic insulin resistance [42]. In addition, elevations of plasma GGT activity are positively associated with increases of visceral adiposity, and this relationship is independent of hepatic fat content [43,44]. It has been suggested that fructose consumption may contribute to increases of hepatic insulin resistance both directly, by providing substrate for hepatic DNL leading to increased triglyceride accumulation and novel PKC activation [6] and indirectly by increasing visceral adiposity [37]. Based on the established associations between GGT activity, increased hepatic lipid content and visceral adiposity it is possible that either or both of these mechanisms may contribute to increases of GGT activity during fructose consumption. However, the association of fructose-induced increases of GGT activity with measures of TG exposure, but not with increases of visceral adiposity, suggests that an increase of intrahepatic lipid may be a primary mechanism. It should also be noted that while increased GGT activity is considered a sensitive marker of increased intrahepatic lipid, it lacks the specificity of other liver enzymes such as ALT. Elevations of GGT activity have also been shown to be associated with increased oxidative stress, all-cause mortality, and mortality from cancer and diabetes as opposed to liver disease alone [45]. It is interesting but not clear why the liver enzymes GGT, ALT and AST decreased significantly from values measured during the baseline complex carbohydrate diet in subjects consuming glucose-sweetened beverages.

Elevations of plasma GGT activity are predictive of the development of metabolic syndrome [14] and it has been suggested that elevated GGT activity should be included as an additional diagnostic risk factor for metabolic syndrome [46]. Our data suggest that under energy balanced conditions, consumption of fructose at 25% of energy requirements for 10 wks leads to greater increases of plasma GGT activity in subjects with less than 3 MSRFs compared to those with metabolic syndrome (≥3 MSRFs), despite comparable baseline values in both groups (see Results). The reasons for this differential response are unclear.

## Conclusions

We report new data demonstrating that consumption of fructose at 25% of energy requirements for 10 wks, when compared with isocaloric consumption of glucose, leads to significant increases of fasting uric acid and 24-h uric acid exposure, as well as circulating concentrations of RBP-4 and GGT activity in overweight/obese adults. The increases of GGT activity and RBP-4 levels in subjects consuming fructose were associated with previously reported increases of TG exposure [6], while increases of fasting uric acid and 24-h uric acid exposure were not. The effect of fructose to increase levels of RBP-4 is much more robust in men than in women and this may be related to the association of these changes with increases of measures of TG exposure, which were also larger in men [6]. Together, the results presented here indicate that prolonged fructose consumption may contribute to the development of the metabolic syndrome by increasing circulating concentrations of uric acid, GGT activity (altered hepatic function), and the production of RBP-4.

## Abbreviations

RBP-4: Retinol binding protein-4; AST: Aspartate aminotransferase; ALT: Alanine aminotransferase; GGT: Gamma-glutamyl transferase; DNL: De novo lipogenesis; TG: Triglyceride; MSRF: Metabolic syndrome risk factor; VAT: Visceral adipose tissue; SAT: Subcutaneous adipose tissue; PKC: Protein kinase C; GLUT4: Glucose transporter-4; VLDL: Very low density lipoprotein.

## Competing interests

None of the authors have any personal or financial conflicts of interest.

## Authors’ contributions

CLC: responsible for organization and analysis of data, primary preparation of the manuscript, and assisted with execution of experimental procedures; KLS: assisted with obtaining funding and design of the study, was responsible for study implementation and supervision, formulation of experimental diets and assisted with manuscript preparation; JLG: assisted with analysis of data and manuscript preparation; JMS: assisted with study design and was responsible for measurement of hepatic DNL; SCG: served as study physician and assisted with design of the study; AAB: served as study physician; LB: assisted with design of the study; NLK: assisted with design and implementation of the study; and PJH: responsible for the conception and design of the study, obtaining funding, and assisted with preparation of the manuscript. All authors read and approved the submitted manuscript.
